# Impact of Junior Doctor-Led Near-Peer Teaching on Medical Student Confidence in Paediatric Prescribing: A Prospective Pre-post Study

**DOI:** 10.7759/cureus.116852

**Published:** 2026-09-24

**Authors:** Safiollah Borhani, Fariha Borhani, Beheshta Borhani, Mehrab Durrani, Zeeshan Ali

**Affiliations:** 1 Hospital Medicine, London North West University Healthcare NHS Trust, London, GBR; 2 Pharmacy, Kenton Bridge Medical Centre, London, GBR; 3 Education, University of Cambridge, Cambridge, GBR; 4 Interventional Radiology, New Cross Hospital, Wolverhampton, GBR; 5 General Practice and Family Medicine, Preston Royal Infirmary, Preston, GBR

**Keywords:** clinical teaching, junior doctors, medical education, medical students, near-peer teaching, paediatric prescribing, prescribing errors, prescribing safety, undergraduate confidence

## Abstract

Background

Paediatric prescribing is a clinical skill that medical students frequently report as an area of low confidence. Near-peer teaching (NPT), facilitated by junior doctors, has demonstrated effectiveness across clinical education settings, but its specific impact on paediatric prescribing confidence has not been previously evaluated in the literature. This study evaluated whether a junior doctor-led NPT programme was associated with improvements in self-reported confidence in paediatric prescribing among Imperial College London medical students.

Methods

A prospective pre-post study was conducted across 12 teaching sessions. Fifty-one students participated, of whom 49 completed both pre- and post-intervention questionnaires. A nine-item, five-point Likert scale instrument assessed four paired domains (prescribing confidence, dose calculation, guideline use, and drug chart preparedness) and five post-only domains (knowledge gain, skill improvement, relevance, near-peer effectiveness, and recommendation). Wilcoxon signed-rank tests were used to compare paired pre- and post-intervention scores.

Results

Statistically significant improvements were observed across all four paired domains (all p < 0.001). Median scores rose from 2.0 (IQR 2-3) pre-intervention to 4.0 (IQR 4-5) post-intervention across all items. Between 95.9% and 100% of students reported improvement in individual items. Post-only items demonstrated strong endorsement: 91.8% of students agreed or strongly agreed that junior doctors were effective and approachable educators, and 83.7% would recommend the sessions to peers.

Conclusion

This study demonstrates that junior doctor-led NPT was associated with substantial improvements in medical students’ self-reported confidence in paediatric prescribing. The findings support the wider integration of structured near-peer programmes into undergraduate paediatric teaching as a scalable, low-cost intervention with meaningful educational impact.

## Introduction

A study in 2010 demonstrated that about 13% of paediatric prescribing contains an error [1]. Prescribing errors are very common and largely preventable, with paediatric prescribing errors being particularly vulnerable. Dosing error was the most common type of medication error, with antibiotics and sedatives being the most common classes of drugs affected, likely because they are the most common paediatric drugs prescribed [2,3].

Paediatric prescribing is often an area of significant anxiety for medical students. A survey of final-year medical students determined that only about 38% of the students felt confident in performing weight-based dose calculations, and only about one in three had written a hospital prescription chart more than three times prior to starting foundation training [4]. These gaps in their confidence and knowledge have significant implications for patient safety [5,6]. A study identified that about 0.4% of prescribed medications in hospital contained a serious error. These were errors that would have resulted in significant harm to patients had they not been intercepted [7].

A widely used method of teaching is near-peer teaching (NPT). It is a model in which learning is facilitated by junior doctors who are at a slightly more advanced stage of training than the learners. Students have described NPT as a more ‘comforting environment’ for learning, and it has been shown to be as effective as qualified tutors in the clinical skills examination [8]. A study by South-east Scotland Foundation Doctors demonstrated that 99% of students who attended NPT expressed interest in attending more tutorials. Furthermore, most of the tutors felt the experience was also useful to them and helped them prepare for teaching [9]. NPT has been shown to benefit the teacher as well as the learner, reinforcing the educator's own clinical knowledge and communication skills [10]. Junior doctors are well placed to deliver teaching to final-year medical students, particularly due to their insight and recent exposure to undergraduate training. This enables them to place themselves in the learners’ position and anticipate the challenges, knowledge gaps, and learning needs that students may encounter. Rees et al. demonstrated that students taught by near-peers reported greater psychological safety when asking questions, a finding particularly relevant to prescribing education, where fear of appearing incompetent may inhibit learning [11].

Studies investigating near-peer prescribing education for medical students have demonstrated the potential benefits of junior doctor-led teaching, including improved learner confidence and skills, as well as a preference for teaching delivered by junior doctors over more senior clinicians [12]. However, there remains limited evidence specifically examining the use of junior doctors to deliver paediatric prescribing education, a distinct and important gap given the additional complexities associated with prescribing for children, including weight-based dosing and age-dependent pharmacokinetics. This study evaluated whether a junior doctor-led NPT programme was associated with improvements in self-reported confidence in paediatric prescribing among undergraduate medical students.

## Materials and methods

Study design

This was a single-centre, prospective pre-post study in which medical students completed a questionnaire before and after the teaching session. The pre-study and post-study questionnaire samples are presented in Appendix A. Data collection was conducted between March 2026 and July 2026. The teaching was delivered by foundation year doctors, General Practice specialty trainee doctors, or paediatric core trainee doctors, all with recent paediatric experience. The teaching session included interactive case-based scenarios. Three to five clinical scenarios were chosen to be discussed over 30 minutes. Cases were designed to reflect common paediatric presentations and required students to select appropriate medications and calculate doses and fluid requirements. Students documented their medication and fluid prescriptions on a drug chart, which was subsequently reviewed by the tutor, and feedback was given, with particular attention to the medication selected, dose, frequency, route, and duration. The prescribing was structured around the British Pharmacological Society’s Principles of Good Prescribing, which provides a framework for safe and effective prescribing [13]. Local prescribing guidelines were also discussed and referenced throughout, ensuring that teaching reflected the protocols students would encounter in their clinical environment. To promote consistency across sessions, all tutors were provided with standardised clinical vignettes (Figure 1), a shared teaching framework, and guidance on feedback delivery prior to their session. Brief orientation was provided to ensure familiarity with the session structure, the BPS Principles of Good Prescribing framework, and the expected approach to drug chart feedback. Only final-year medical students or students in their penultimate year were included. Each teaching session aimed to have a maximum of eight students to maximise the value of education. Participation was voluntary.

**Figure 1:**
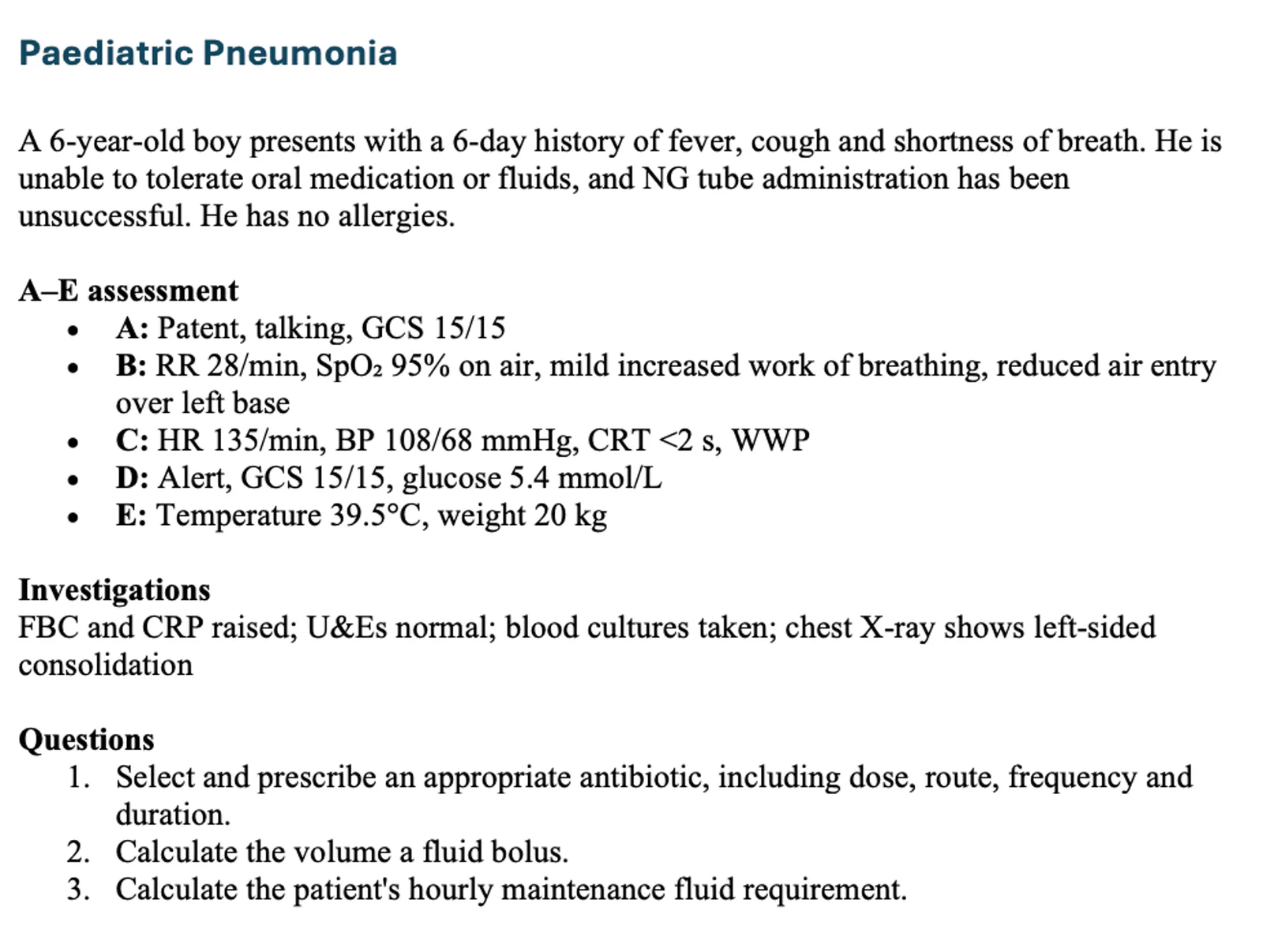
Clinical Vignette Example of a standardised clinical vignette provided to tutors prior to each teaching session. A-E: Airway, Breathing, Circulation, Disability, Exposure; A&E: Airway and Exposure; AVPU: Alert, Voice, Pain, Unresponsive; BP: Blood Pressure; BNFc: British National Formulary for Children; bpm: Beats per Minute; CRP: C-Reactive Protein; CRT: Capillary Refill Time; FBC: Full Blood Count; GCS: Glasgow Coma Scale; HR: Heart Rate; IQR: Interquartile Range; IV: Intravenous; RR: Respiratory Rate; SpO₂: Oxygen Saturation; TDS: Three Times Daily; U&E: Urea and Electrolytes; WBC: White Blood Cell Count

Outcome measures

The outcome was assessed by a self-reported questionnaire that was completed before and after the teaching session. The items were grouped into four domains: Confidence; Knowledge & Skills; Usefulness & Feedback; and Near-Peer Perception. The questionnaire collected the data via a five-point Likert response option, ranging from 1 (“strongly disagree”) to 5 (“strongly agree”). Items 1 to 4 were filled out before and after the teaching to show changes in confidence and knowledge & skills. Items 5 to 9 were all completed after the teaching session only. The questionnaire is demonstrated in Table 1 and Table 2. The aim of the questionnaire was to determine the confidence and prior experience of medical students. It also demonstrated to tutors what areas of paediatric prescribing medical students struggled with and required further training. The questionnaire was reviewed by the teaching team prior to use to ensure content validity and alignment with the learning objectives of the sessions. The questionnaire was purpose-designed following a review of the existing literature on undergraduate prescribing education and NPT. Items were selected to reflect the key domains of paediatric prescribing competence identified as areas of greatest difficulty for undergraduate students, including dose calculation, guideline application, and drug chart completion [3,4]. Self-reported confidence was chosen as the primary outcome measure. A five-point Likert response scale was selected as it is widely used in medical education research and is appropriate for ordinal data analysis using non-parametric statistical methods. Formal psychometric validation, including assessment of internal consistency and test-retest reliability, was not undertaken given the service evaluation context of this study.

**Table 1:** Pre-study Questionnaire Items

No.	Item	Domain
1	I feel confident in prescribing for paediatric patients	Confidence
2	I feel capable of calculating accurate drug doses for children	Knowledge and Skills
3	I am comfortable using local and national guidelines to prescribe medications for children	Knowledge and Skills
4	I feel prepared to write or check a paediatric drug chart	Knowledge and Skills

**Table 2:** Post-study Questionnaire Items

No.	Item	Domain
1	I feel confident in prescribing for paediatric patients	Confidence
2	I feel capable of calculating accurate drug doses for children	Knowledge and Skills
3	I am comfortable using local and national guidelines to prescribe medications for children	Knowledge and Skills
4	I feel prepared to write or check a paediatric drug chart	Knowledge and Skills
5	The tutorial enhanced my knowledge of safe paediatric prescribing	Usefulness and Feedback
6	The tutorial improved my practical prescribing skills	Usefulness and Feedback
7	The content of the tutorial was relevant and useful to my clinical learning	Usefulness and Feedback
8	Junior doctors are effective and approachable educators for paediatric prescribing	Near-Peer Perceptions
9	I would recommend the tutorials to other medical students	Near-Peer Perceptions

Statistical analysis was performed using IBM SPSS Statistics for Windows, Version 29.0 (Released 2022; IBM Corp., Armonk, NY, USA). Wilcoxon signed-rank tests were used for paired comparisons. Test statistics (W) and exact p-values are reported. Effect size was calculated using $r = Z/\sqrt{N}$, where Z is the standardised test statistic, and N is the total number of observations. As this was a pilot educational evaluation, a formal a priori sample size calculation was not performed. The target sample was determined pragmatically based on the expected number of students attending over the study period. 

The study was submitted to the Education Ethics Review Process (EERP) at Imperial College London for ethical classification. Following review, the EERP team stated that the study did not require formal ethical approval. This decision was made on the basis that the study was designed to assess the effectiveness of a local teaching intervention for quality improvement purposes rather than to test educational research. All participation was voluntary and anonymous. No patient data were collected at any point. Participants provided implied consent by completing and returning the questionnaire. Data were stored securely and used solely for the purposes of this evaluation.

## Results

Participants

A total of 51 medical students participated across 12 teaching sessions, with an average of 4.25 students per session. Forty-nine students (96.1%) completed the pre- and post-teaching questionnaire. These students were included in the paired analysis. Two students who attended the sessions did not complete both the pre- and post-intervention questionnaires and were therefore excluded from the paired analysis. Thirty-three students (64.7%) were in their final year, and 18 students (35.3%) were in their penultimate year of medical school. No patient or student data were collected, and no demographics beyond the year of study were recorded.

Items 1-4

Statistically significant improvements were observed across all four paired domains following the teaching intervention, reflecting improvements in self-reported confidence and perceived prescribing skills.

Prescribing confidence (Item 1) showed an increase in median score from 2.0 (IQR 2-3) to 4.0 (IQR 4-5), with 47 of 49 students (95.9%) reporting an improvement. Confidence in dose calculation (Item 2) improved in 48 of 49 students (98.0%), with median scores rising from 2.0 (IQR 2-3) to 4.0 (IQR 4-5). All 49 students (100%) reported improvement in comfort using local and national guidelines (Item 3), with a pre-intervention median of 2.0 (IQR 2-3) rising to 4.0 (IQR 4-5) post-intervention. Preparedness to write or check a paediatric drug chart (Item 4) showed improvement in 48 of 49 students (98.0%), with a median increase from 2.0 (IQR 2-3) to 4.0 (IQR 4-5). All four improvements were statistically significant on Wilcoxon signed-rank testing (p < 0.001 for all). Results are illustrated in Figure 2.

**Figure 2:**
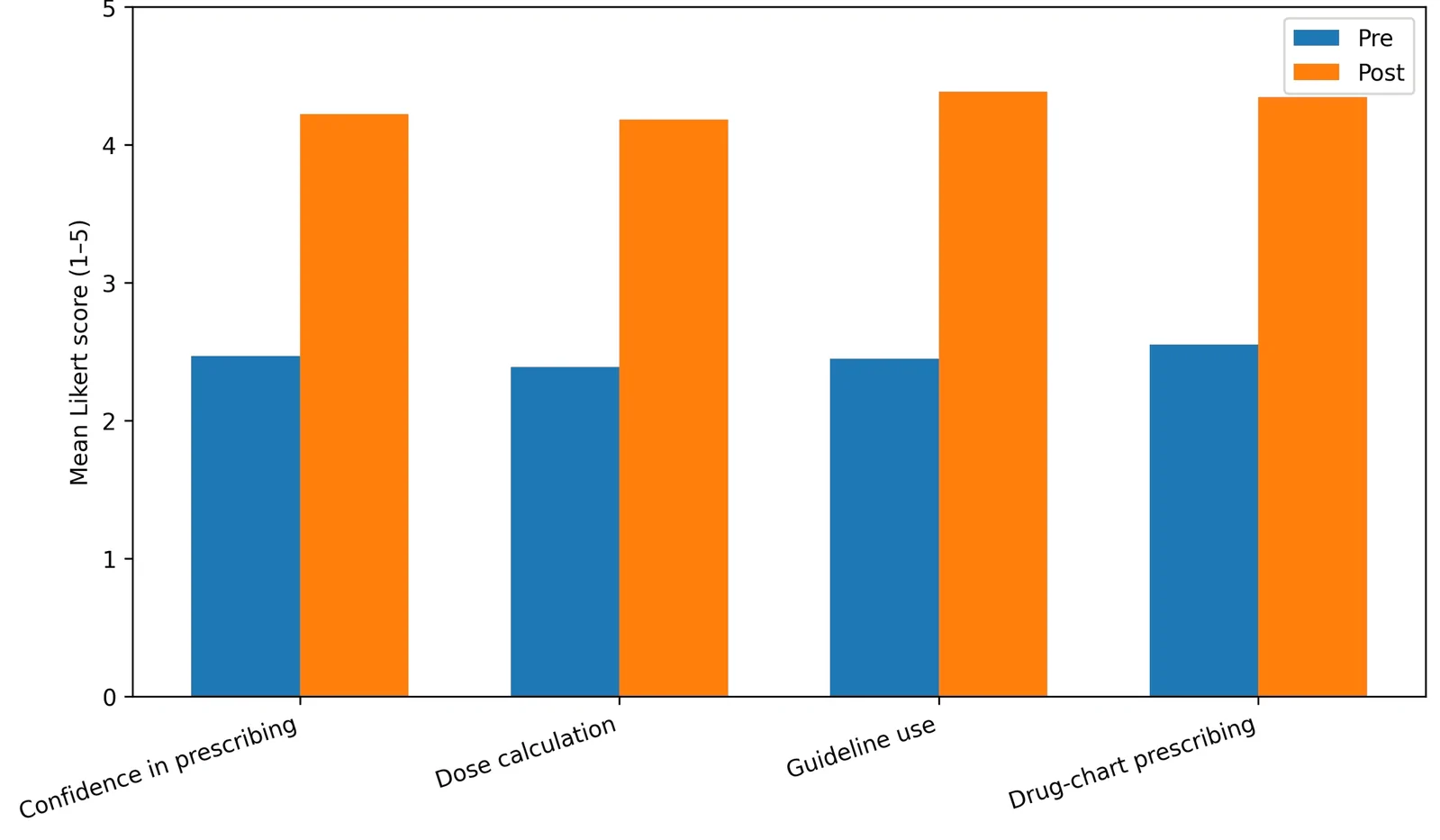
Mean Likert Score Pre- and Post-teaching Mean Likert scores for Items 1-4 pre- and post-teaching intervention. Items assessed prescribing confidence, dose calculation, guideline use, and drug chart preparedness, respectively. Scores range from 1 (strongly disagree) to 5 (strongly agree). All comparisons reached statistical significance (p < 0.001, Wilcoxon signed-rank test). Pre = pre-teaching; Post = post-teaching.

The values of the effect sizes are as follows: Item 1: W = 0, Z = -5.98, r = 0.85 (large effect); Item 2: W = 0, Z = -6.01, r = 0.86 (large effect); Item 3: W = 0, Z = -6.08, r = 0.87 (large effect); Item 4: W = 0, Z = -6.01, r = 0.86 (large effect).

Items 5-9

Post-intervention responses to items assessing the educational value of the sessions were strongly positive. Regarding knowledge of safe paediatric prescribing (Item 5), 44 of 49 students (89.8%) agreed or strongly agreed that the tutorial enhanced their knowledge, with a median score of 5.0 (IQR 4-5). Perceived improvement in practical prescribing skills (Item 6) was reported by 46 of 49 students (93.9%), with a median score of 5.0 (IQR 4-5). This was the highest endorsement of any item. The relevance and usefulness of session content to clinical learning (Item 7) was affirmed by 40 of 49 students (81.6%), with a median score of 5.0 (IQR 4-5). Students reported highly favourable perceptions of junior doctors as educators. Regarding their effectiveness and approachability as educators for paediatric prescribing, 45 of 49 students (91.8%) agreed or strongly agreed (Item 8), with a median score of 5.0 (IQR 4-5). Regarding their willingness to recommend the tutorials to fellow medical students, 41 of 49 students (83.7%) stated that they would do so (Item 9), with a median score of 5.0 (IQR 4-5). Results are illustrated in Figure 3.

**Figure 3:**
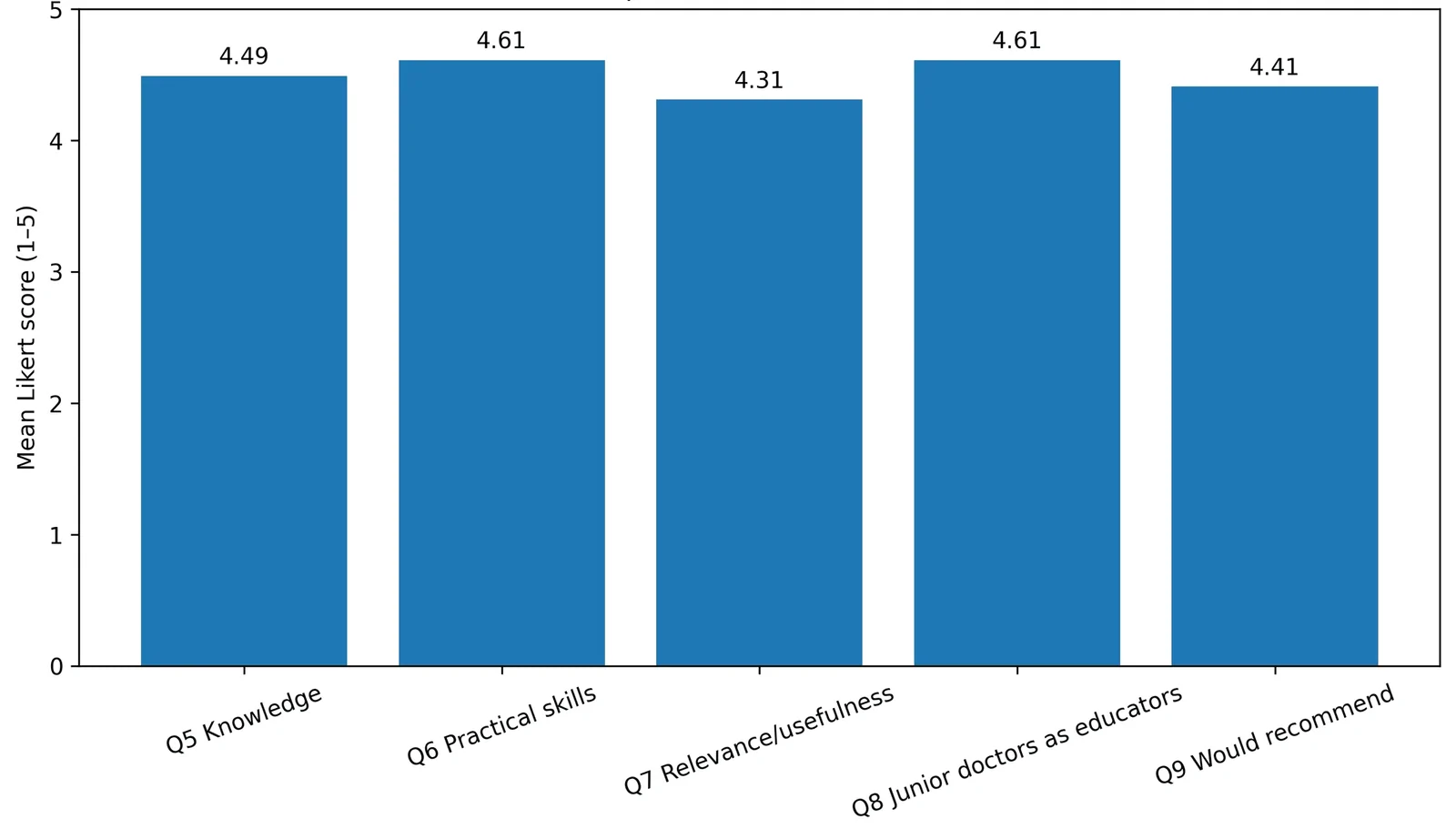
Mean Likert Score Post-teaching Mean Likert scores for post-intervention items (Items 5-9). Items assessed knowledge gain, practical prescribing skills, relevance of content, near-peer teaching effectiveness and likelihood of recommendation, respectively. Scores range from 1 (strongly disagree) to 5 (strongly agree).

## Discussion

This study evaluated the impact of a junior doctor-led NPT programme on medical student confidence in paediatric prescribing. Across 12 sessions, improvements in self-reported confidence were demonstrated across all four paired domains. Between 95.9% and 100% of students reported improvement after a single session. Post-intervention responses demonstrated high levels of satisfaction.

Several strengths of this study merit recognition. The prospective pre-post design with paired measurements enabled direct comparison of pre- and post-intervention scores within the same cohort. The use of standardised clinical vignettes and a shared teaching framework across all 12 sessions promoted consistency of the intervention. The reporting of effect sizes alongside p-values provides a more complete picture of the magnitude of change observed. The small-group format facilitated individualised feedback and active participation.

The impact of the teaching intervention was notable. Pre-intervention median scores across all four of the paired items were 2.0, demonstrating that students’ confidence in paediatric prescribing was low. This is a finding that has been consistent across other research on medical students’ prescribing [4,14,15]. However, following the teaching session, the median score across the paired domains improved to 4. This is particularly noteworthy given previous concerns regarding the preparedness of medical students for prescribing tasks during the transition to clinical practice [16]. Notably, 100% of students reported improved confidence in using local and national prescribing guidelines (Item 3). This finding suggests that a structured, case-based teaching approach incorporating practical use of the British National Formulary for Children (BNFc) and local prescribing guidelines may have a substantial positive effect on students’ confidence in applying prescribing resources. Previous studies have demonstrated a similar need for further prescribing education among medical students, particularly in the application of prescribing guidelines and BNFc to improve students’ confidence and preparedness to prescribe [17,18].

These findings are consistent with established evidence supporting NPT as an effective educational approach. Previous research suggests that NPT may enhance learning through cognitive congruence, whereby teachers, having recently experienced similar educational and clinical challenges, may be better positioned to understand learners’ difficulties and facilitate the transition from theory to clinical practice [19]. This is particularly relevant to paediatric prescribing, an area in which newly qualified doctors have been reported to feel underprepared [4].

This theory is supported by more than 91% of the medical students who agreed that junior doctors were effective and approachable educators for paediatric prescribing. Compared with consultant-led teaching, NPT may reduce perceived hierarchical barriers, potentially encouraging students to participate more actively and ask questions, particularly when discussing complex topics such as paediatric prescribing. Furthermore, more than 80% of students reported benefiting from the session and indicated that they would recommend it to their peers, supporting the acceptability and perceived value of junior doctor-led NPT.

A further notable finding was the high endorsement that was observed for Item 6, with more than 93% of students reporting improved practical prescribing skills following the session. This finding supports the potential value of case-based teaching in developing students’ confidence and perceived prescribing skills. Previous research has similarly demonstrated that case-based learning can improve students’ knowledge and confidence when compared with more traditional teaching approaches [20,21]. The integration of the BPS Principles of Good Prescribing provided students with a structured framework for approaching prescribing decisions.

The small-group format adopted in this study, with an average of 4.25 students per session, aligns with evidence suggesting that smaller group sizes facilitate deeper engagement and greater confidence in asking questions [22].

Limitations

There are several limitations of our study that need to be acknowledged. First, although improvements were observed in self-reported confidence, this does not directly measure prescribing competence or error rate. However, Bandura's social cognitive theory proposes that an individual's belief in their capacity to perform a specific task is a reliable predictor of subsequent performance and behaviour [23]. Applied to prescribing education, improvements in prescribing self-efficacy following a structured intervention may therefore translate into greater engagement with prescribing tasks during foundation training. Future work should seek to incorporate objective assessments of the prescribing skills of medical students pre- and post-teaching. Second, longer-term follow-up would have been beneficial to assess the durability of the confidence gained. Whether improvements in confidence are sustained over weeks and months was beyond the scope of this evaluation. Third, whilst the anonymous study design is ethically appropriate, it did not take into account any previous paediatric prescribing teaching or clinical exposure. Students who had already attended similar sessions, completed a prior paediatric placement, or received prescribing education may have entered the intervention with a higher baseline confidence, potentially influencing both pre- and post-intervention scores. Although standardised vignettes and a shared teaching framework were used, the teaching was delivered by different tutors across 12 sessions. This could have led to variability in teaching style and clinical experience of the tutors, which may have led to inconsistency in the learning experience. The absence of a control or comparison group is a significant methodological limitation. Without a control group, it is not possible to exclude the possibility that improvements in self-reported confidence reflect natural progression rather than a direct effect of the teaching intervention, which substantially limits causal inference. The small, single-centre sample of 49 students restricts the generalisability of findings to other institutions, clinical settings, and student cohorts. Voluntary participation may have also introduced selection bias, as students who chose to engage may have had greater motivation or interest in paediatric prescribing than those who did not. Finally, the questionnaire was not formally validated, and its psychometric properties have not been established, which limits the rigour of the outcome measurement.

Future directions

Future research should address the limitations identified in this study. Multicentre, randomised controlled studies with larger sample sizes and a control group would strengthen the evidence base and allow for more robust causal conclusions. The inclusion of objective prescribing assessments, such as the Prescribing Safety Assessment or observed structured clinical examinations, alongside self-reported measures would provide a more comprehensive evaluation of the intervention's impact on prescribing competence. Open-ended qualitative feedback from students would complement the quantitative data and provide richer insight into the learning experience. Longitudinal follow-up studies are needed to determine whether improvements in confidence are sustained over time and whether they translate into reduced prescribing error rates during foundation training.

## Conclusions

Junior doctor-led NPT, delivered through focused teaching sessions and clinical case-based scenarios, was associated with substantial and statistically significant improvements in medical students' self-reported confidence in paediatric prescribing. The intervention was highly acceptable to students, with the majority endorsing junior doctors as effective and approachable educators and indicating they would recommend the programme to peers. These findings support the integration of NPT into undergraduate paediatric curricula as a practical and educationally effective approach to addressing a recognised gap in prescribing preparation. Given the established link between undergraduate prescribing preparation and foundation year prescribing error rates, early targeted interventions of this kind may hypothetically carry implications for patient safety, though this remains to be demonstrated in future research. Larger, controlled, multicentre studies including objective prescribing assessments and qualitative outcome measures are warranted to confirm and build upon these results.

## Appendices

Appendix A

Sample of Pre-study Questionnaire

1. I feel confident in prescribing for paediatric patients 

2. I feel capable of calculating accurate drug doses for children 

3. I am comfortable using local and national guidelines to prescribe medications for children 

4. I feel prepared to write or check a paediatric drug chart 

Sample of Post-study Questionnaire

1. I feel confident in prescribing for paediatric patients 

2. I feel capable of calculating accurate drug doses for children 

3. I am comfortable using local and national guidelines to prescribe medications for children 

4. I feel prepared to write or check a paediatric drug chart 

5. The tutorial enhanced my knowledge of safe paediatric prescribing 

6. The tutorial improved my practical prescribing skills 

7. The content of the tutorial was relevant and useful to my clinical learning 

8. Junior doctors are effective and approachable educators for paediatric prescribing 

9. I would recommend the tutorials to other medical students 
